# Regulation of Sox6 by Cyclin Dependent Kinase 5 in Brain

**DOI:** 10.1371/journal.pone.0089310

**Published:** 2014-03-24

**Authors:** Parvathi Rudrabhatla, Elias Utreras, Howard Jaffe, Ashok B. Kulkarni

**Affiliations:** 1 Laboratory of Neurochemistry, National Institute of Neurological Disorders and Stroke, National Institutes of Health, Bethesda, Maryland, United States of America; 2 Laboratory of Neurobiology, National Institute of Neurological Disorders and Stroke, National Institutes of Health, Bethesda, Maryland, United States of America; 3 Protein/Peptide Sequencing Facility, National Institute of Neurological Diseases and Stroke, National Institutes of Health, Bethesda, Maryland, United States of America; 4 Functional Genomics Section, Laboratory of Cell and Developmental Biology, National Institute of Dental and Craniofacial Research, National Institutes of Health, Bethesda, Maryland, United States of America; University of Hong Kong, Hong Kong

## Abstract

Cyclin dependent kinase 5 (Cdk5) is a proline-directed Ser/Thr kinase involved in various biological functions during normal brain development and neurodegeneration. In brain, Cdk5 activity is specific to post-mitotic neurons, due to neuronal specific expression of its activator p35. The biological functions of Cdk5 have been ascribed to its cytoplasmic substrates, however not much is known in nucleus. Here, we show that nuclear transcription factor Sox6 is a direct nuclear target of Cdk5. Sox6 is expressed in Tuj1 positive neurons, suggesting that Sox6 is expressed in differentiating neurons. The expression of Sox6 is high in mitotic nuclei during embryonic day 12 (E12) and gradually decreases during development into adult. On the other hand, Cdk5 expression gradually increases during its development. We show that Sox6 is expressed in mitotic nuclei in embryonic day 12 (E12) and in migrating neurons of E16. Sox6 is phosphorylated *in vivo*. Sox6 was detected by phospho-Ser/Thr and phospho-Ser/Thr-Pro and MPM-2 (Mitotic protein #2) antibodies in brain. Furthermore, calf intestinal alkaline phosphatase (CIAP) digestion resulted in faster migration of Sox6 band. The GST-Sox6 was phosphorylated by Cdk5/p35. The mass spectrometry analysis revealed that Sox6 is phosphorylated at T^119^PER motif. We show that Sox6 steady state levels are regulated by Cdk5. Cdk5 knockout mice die *in utero* and Sox6 protein expression is remarkably high in Cdk5−/− brain, however, there is no change in mRNA expression, suggesting a post-translational regulation of Sox6 by Cdk5. Transfection of primary cortical neurons with WT Cdk5 reduced Sox6 levels, while dominant negative (DN) Cdk5 and p35 increased Sox6 levels. Thus, our results indicate that Cdk5 regulates Sox6 steady state protein level that has an important role in brain development and function.

## Introduction

When metabolic pathways vital for development go awry, the consequences can be disastrous. Sox [Sry-related high-mobility-group (HMG) box] family of transcription factors play an important role in development and are involved in various physiological processes. Twenty Sox transcription factors (Sox1-20) exist in mice and humans [1], [2]. They feature a Sry-related high-mobility-group (HMG) box DNA-binding domain. Sox6 is highly identical to Sox5 and Sox13, forming with them the Sox D subfamily. *Sox6* is known to be highly expressed in neuronal cells, chondrocytes, notochord and spermatid cells, and expressed weakly in muscle cells [3], [4]. Sox5^−/−^Sox6^−*/−*^ fetuses develop severe skeletal dysplasia [5]. About half die at birth and the others fail to thrive after postnatal day 7 (P7) and die at approximately P14. The cause of death remains unclear. Mice with a chromosomal inversion (*p^100H^*) disrupting Sox6 have the same gross phenotype as Sox6^−/−^ mice. They were shown to develop cardiac and skeletal myopathy, suggesting that Sox6 might promote myocyte maturation [6]. Interestingly, Sox6 is prominently expressed in mouse glioma [7]. Sox6 is necessary for normal positioning and maturation of medial ganglionic eminences (MGE) derived cortical interneurons. As a consequence, the specific removal of Sox6 from this population results in a severe epileptic encephalopathy [8]. The microRNA-219 (miR-219) is shown to inhibit oligodendrocyte formation through repression of Sox6 [9].

The proline-directed serine/threonine protein kinase Cdk5, together with its neuronal-specific activating cofactors p35 and p39 have been implicated in many normal and pathological processes in the mammalian central nervous system (CNS) [10], [11]. Cdk5 is also implicated in numerous complex functions of the adult CNS such as synaptic transmission, synaptic plasticity, and neuronal signaling [12]. We have earlier reported neuronal migration defects in Cdk5^−/−^ brain resulting in developmental abnormalities and early lethality [13]–[15]. Cdk5 regulates many cellular processes and functions in the brain, and its deregulation via the calpain-dependent cleavage of its activator p35 to p25 has been implicated in neurodegenerative disorders such as Alzheimer's and Parkinson's diseases [16], [17]. Recently, Cdk5 has been shown to play an important role in regulating transcription factors and chromatin remodeling. Transcription factor MEF2 is shown to be regulated by Cdk5 during neurotoxicity induced apoptosis [18]. Furthermore, Cdk5 mediated degradation of MEF2D is known to be mediated by chaperone induced autophagy [19]. Deregulation of histone deacetylase 1 (HDAC1) activity by Cdk5/p25 induces aberrant cell-cycle activity and double-strand DNA breaks leading to neurotoxicity [20]. The nuclear Cdk5 plays an active role in allowing neurons to remain post mitotic as they mature and that loss of nuclear Cdk5 leads to cell cycle entry [21]. The role of Sox6 in the mammalian brain has not been explored in detail. Since Cdk5 is developmentally regulated [22] and Sox6 has potential Cdk5 phosphorylation sites, it would be of interest to find out whether Sox6 is developmentally regulated by Cdk5?

Here, we report that Sox6 is expressed during early embryonic development and its expression is gradually decreased during its development into adult. Cdk5^−/−^ mice show increased Sox6 expression and inhibition of Cdk5 activity in the primary cortical neurons by DN Cdk5 increases the endogenous Sox6 expression.

## Materials and Methods

### Chemicals and Antibodies

Sox6 and Cdk5 antibodies were purchased from Santa Cruz Biotechnology (Santa Cruz, CA, USA). Tuj1 and β-actin antibodies were purchased from Sigma chemical company (St. Louis, Missouri, USA). Inhibitors such as roscovitine were purchased from Calbiochem (EMD chemicals, Gibbstown, NJ, USA).

### Animals

Cdk5-knockout mice were generated as previously reported [22]. All of the animal procedures were conducted in accordance with the National Institutes of Health guidelines for the care and use of laboratory animals. The animal procedures were approved by National Institute of Neurological Disorders and Stroke (NINDS) Animal Care and Use Committee (ACUC), NIH. This study was carried out in strict accordance with the recommendations in the Guide for the Care and Use of Laboratory Animals of the National Institutes of Health. The protocol was approved by the Committee on the Ethics of Animal Experiments of the Animal Care and Use Committee (ACUC), National Institute Neurological Disorders and Stroke (NINDS), NIH.

#### *In Vitro* Kinase Assays

The *in vitro* kinase assay was performed with either purified recombinant Sox6 protein (0.5 µg) or Sox6 immunoprecipitated from rat brain lysate or from primary cortical cultures. Sox6 protein was incubated with purified Cdk5/p35 complex in a kinase reaction buffer containing [γ-^32^P] and cold ATP (50 µM). For some experiments Cdk5/p35 complex was immunoprecipitated from the primary cortical neurons. The kinase reaction was carried out for 30 min at 30°C and terminated by the addition of Laemmli sample buffer. Reaction products were resolved by SDS-PAGE, and ^32^P-labeled proteins were visualized by autoradiography.

### Culture of Primary Cortical Neurons

Primary cortical neurons were established from embryonic day-18 (E18) Sprague–Dawley rat embryos (Charles River Labs, NY, USA). An eighteen day timed pregnant rat was euthanized using CO_2_ and pups were removed, decapitated and the cortex was dissected in Hibernate-E media (Brain Bits LLC, IL, USA). Dissociated cortical neurons were obtained by incubating the cortex in EBSS containing 15 units/mL of papain (Worthington Biochemicals, NJ, USA) for 45 min at 37°C before triturating in neurobasal medium containing 20% fetal bovine serum (Hyclone, UT, USA), DNAse (0.2 mg/mL). Undissociated neurons were removed from the cell suspension by passing the cell suspension through a 40 µm cell strainer (Fisher Scientific, NY, USA). Neurons were centrifuged at 2000 *g* for 3 min at 20°C and the pellet was resuspended in neurobasal medium supplemented with B27, penicillin (100 U/mL), streptomycin (100 U/mL) and L-glutamine (0.5 mM, Invitrogen, NY, USA). Neurons were then plated at a density of 150 000 cells/mL on circular glass coverslips and 6-well tissue culture dishes, coated with poly-L-lysine (50 µg/mL, Sigma Chemicals, MO, USA), and incubated in a humidified atmosphere containing 5% CO_2_: 95% O_2_ at 37°C.

### RNA Isolation and Real Time PCR

Total RNA was isolated from the WT, Cdk5^−/−^, p35^−/−^ using TRIzol reagent (Invitrogen) according to the manufacturer's instructions. Following TURBO DNA-free (Ambion, Austin, TX, USA) digestion of total RNA sample, to remove contaminated genomic DNA, oligo(dT) primed synthesis of cDNA from 1 µg of total RNA was made using Super-Script III reverse transcriptase (Invitrogen). The PCR consisted of 35 cycles of 30 s each at 94°C, 60°C, and 72°C. For detection of Sox6, p35, and Cdk5 mRNA we used real time PCR, and the following reaction mixture was used for these PCR samples: 1×IQ Sybr Green Super Mix (Bio-Rad, Berkeley, California. USA), 100–200 nM of each primer and 1 µl of cDNA. cDNA was amplified and analyzed in triplicate using Opticon Monitor Chromo 4 (Bio-Rad). The following primers were used to amplify and measure the amount of mouse mRNA by real time reverse transcription-PCR: Sox6 S: 5′-CATGTCCAACCAGGAGAAGCA-3′ Sox6 AS: 5′GGGTACTTCTCTAGGTGGATTTTGC-3′, Cdk5 S: 5′-GGC TAA AAA CCG GGA AAC TC-3′ and Cdk5 AS: 5′-CCA TTG CAG CTG TCG AAA TA-3′ [23]. The mRNA levels were standardized by using the following primers to GAPDH: GAPDH S: 5′-AAT GTG TCC GTC GTG GAT CTG A-3′ and GAPDH AS: 5′-GAT GCC TGC TTC ACC ACC TTC T-3′.

### CIAP assay

The 25 µg of the brain lysate was subjected to 1 unit of calf intestinal alkaline phosphatase (CIAP) and the 25 µg of untreated and treated lysates were run on SDS-PAGE gel. Western blotting was performed with Sox6 antibody.

### Co-immunoprecipitation assay

For co-immunoprecipitation of endogenous proteins, neurons were lysed in ice-cold buffer containing 50 mM Tris–HCl (pH 7.5), 150 mM NaCl, 10 mM EDTA, 2 mM EGTA, 1% NP-40, supplemented with the phosphatase and protease inhibitors cited above. Cell extracts were clarified by centrifugation and supernatants (500 µg protein) were incubated with anti-Cdk5 (2 µg) overnight at 4°C, followed by the addition of 30 µl of protein A-agarose (GE Healthcare Life Sciences, Uppsala, Sweden), for 2 h at 4°C. Supernatants (50 µg protein) were incubated with anti-Sox6 or anti-Cdk5 or anti-p35 in the presence of 10 µl of protein A-agarose. Inmunoprecipitates were extensively washed with lysis buffer and detected by Western blot analysis. NIH ImageJ software was used to analyze and quantitate the bands in Western blots.

### Protein estimation and Western Blotting

Protein estimation was performed according to BCA method. Western blotting was performed as previously described [23], [24].

### Immunocytochemistry of primary cortical neurons

Staining of cortical neurons was performed as described earlier [25], [26]. Cortical neurons fixed in 4% paraformaldehyde were permeabilized for 20 min in 0.2% Triton X-100, blocked in 10% horse serum in phosphate-buffered saline (PBS) for 1 h, and incubated with primary antibodies (diluted in 4% horse serum/phosphate buffered saline 0.05% Tween-20) against primary antibody for overnight at 4°C. After three washes in wash solution (phosphate buffered saline 0.05% Tween-20), the neurons were incubated with anti-rabbit (Alexa 488) and anti-mouse (Alexa 568) secondary antibodies (Molecular Probes, Eugene, OR, USA). Secondary antibodies were diluted in the same buffer as the primary antibodies and incubated for 2 h at room temperature. Cells were washed and mounted on the cover slips and analyzed using a TCS software program on an Axiovert 200 M microscope (Zeiss, Germany).

### Immunohistochemical staining of developmental brain sections

Paraffin-embedded embryonic brain sections were prepared for immunostaining through xylene treatment and gradual rehydration with 95–75% ethanol. Sections were blocked (5% goat serum/0.1% Triton X-100) and then incubated with primary antibodies (Sox6 antibodies) overnight at 4°C in blocking solution. The sections were incubated with peroxidase conjugated secondary antibodies for 1 h at room temperature. The sections were stained using 3,3′ diaminobenzidine and counterstained with methylene blue, and slides were coverslipped using Permount (Biomeda, Foster City, CA, USA). Images were captured with a ×40 objective on a Nikon Eclipse E400 microscope using SPOT software (Nikon, Tokyo, Japan).

#### In vitro phosphorylation of Sox6 by Cdk5 and subsequent mass spectrometry analyses

Culture (∼1 liter) of the BL21 bacteria transformed with pGEX-4T3-Sox6 protein (GST-Sox6) was purified by using the Glutathione Sepharose GSTrap 4B columns (GE Healthcare Bioscience Corp.). The purified GST-Sox6 protein was mixed with 10 ng of active CDK5/p35 (Millipore Corp., Billerica, MA) in a buffer containing 20 mM HEPES (pH 7.4), 1 mM EDTA, 10 mM MgCl_2_, 0.2 mM dithiothreitol, and a protease inhibitor cocktail (2 µl) in a final volume of 50 µl. The reaction was started by addition of 100 µM ATP and incubated at 30 C for 30 min. The samples were buffer exchanged into 25 mM NH_4_HCO_3_ by using 30K Nanosep Centrifugal Devices (Pall Corp., East Hills, NY, USA). They were then dried in the SpeedVac System (ThermoSavant Corp., Holbrook, NY, USA) and were taken up in 8 M urea/0.4 M NH_4_HCO_3_ for reduction (by dithiothreitol) and alkylation (by iodoacetamide) according to the standard protocol [27]. After diluting the samples into 2 M urea/0.1 M NH_4_HCO_3_ with water, half of each sample was digested with trypsin and half with chymotrypsin overnight at 37°C. Digested samples were then acidified with trifluoroacetic acid, combined, and cleaned up in the OASIS HLB media (Waters Corp., Milford, MA, USA). Eluted digests were dried in the SpeedVac system and were subjected to enrich the phosphorylated peptides by the TiO_2_ chromatography, according to the method of Wu *et al.* [28]. Phosphopeptide-enriched samples were finally analyzed with liquid chromatography/tandem mass spectroscopy (MS/MS) on an LTQ XL mass spectrometer (Thermo Electron Corp., San Jose, CA, USA) where the instrument was set up to acquire a full survey scan followed by a collision-induced dissociation MS/MS spectrum on each of the top 10 most abundant ions in the survey scan. MS/MS Spectra were searched by using the SEQUEST program (Thermo Electron, Inc.) against a FASTA database, which consists of sequences of the pertinent protein and several decoy protein to identify phosphopeptides and specific phosphorylation sites.

### Statistical analysis

Measurements from individual experiments were always performed in triplicate. The results are expressed as mean±SD. Statistical analysis of the results was performed by student's t-test and *p*<0.05 level was considered as satistically significant.

## Results

### Sox6 is expressed in post mitotic neurons

**Figure 1:**
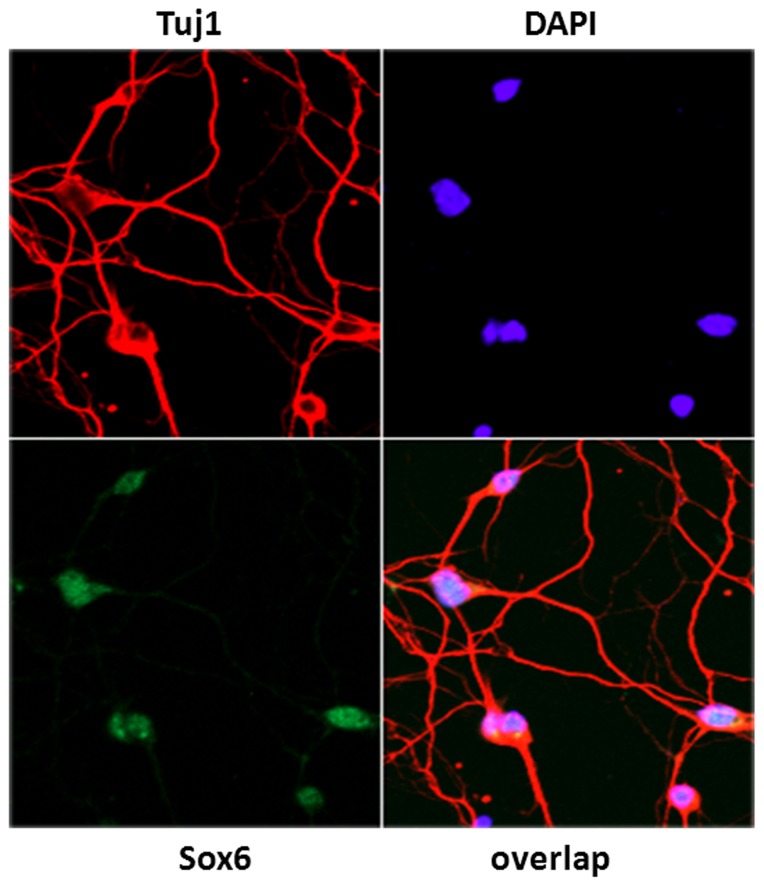
Sox6 is expressed in post-mitotic neurons and inhibition of Sox6 results in neurite retraction and apoptosis. Embryonic day 17 (E17) dissociated rat cortical neurons were cultured *in vitro* for 7 days. Cells were fixed and immunostained with a Sox6-specific antibody (green) and an antibody to TuJ1 (red), a marker for differentiating neurons. Sox6 is robustly expressed in nuclei of differentiating neurons. Nuclei were counterstained using DAPI.

To examine if Sox6 is expressed in differentiating neurons, cortical neurons prepared from embryonic day 17 (E17) dissociated rat cortex were immunostained for TuJ1, a marker for differentiating neurons and Sox6. Cells expressing the neuron-specific marker TuJ1 (red) were also found to express Sox6 (green). The Sox6 protein was detected in the nucleus in the TuJ1 positive neurons, suggesting that Sox6 is expressed in differentiating neurons (Figure 1).

### Sox6 is developmentally regulated in brain

**Figure 2:**
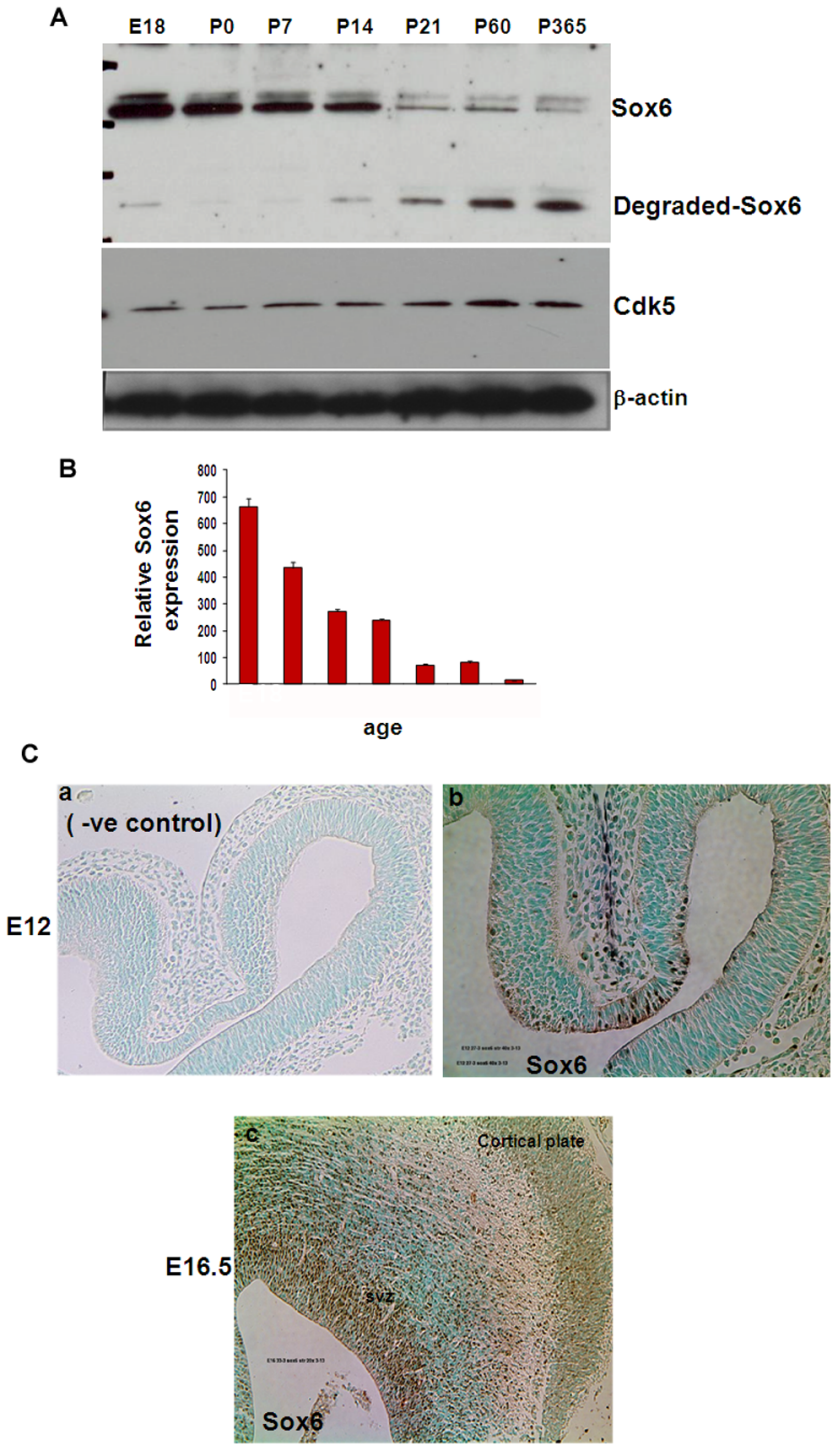
Sox6 is developmentally regulated in brain. A) Developmental expression of Sox6 (upper panel) and Cdk5 (middle panel) in embryonic brain (E18), postnatal days 0, 7, 14, 21, 60 and 365 days. The lower panel shows the β-actin staining as loading control. B) Densitometry analysis of Sox6 obtained from Figure 2A. C) Sections of mouse brain during embryonic development were fixed and immunostained with Sox6-specific antibodies from embryonic day 12, E12 (b) and E16 (c). C*a*, Negative control shows the section of rat brain (E12) that were fixed and immunostained with Sox6 antibodies preabsorbed with Sox6 protein. Immunostaining with pre-immune serum as well as with no primary antibody showed no signal. *Cb*, The E12 rat brain section was fixed and immunostained with Sox6-specific antibodies. Sox6 staining is observed in the mitotic nuclei of E12 rat brain. C*c*, Sox6 staining in the E16 brain. The neocortex-cortical plate and the sub ventricular zone-neuroepithelium (svz) are shown in E16.

To investigate the role of the Sox6 protein during mammalian central nervous system (CNS) development, we first characterized the expression of Sox6 protein in the rodent brain by immunoblotting and immunohistochemistry with the Sox6 antibody. Immunoblotting revealed that the Sox6 is detected in the embryonic brain and its expression gradually decreases during its development into an adult stage (Figure 2A and B). From the immunoblotting analysis, it appears that during development, Sox6 is degraded in the adult stage as Sox6 antibodies recognized a degradation product of the lower molecular weight. On the other hand, Cdk5 levels gradually increased during mouse brain development from E18 until adult (365 days, 1 year) (Figure 2A, middle panel). These results suggest that Sox6 and Cdk5 activity are inversely related. This also raises an intriguing possibility that Sox6 could be phosphorylated and degraded by Cdk5 during development.

Immunohistochemistry analysis revealed that Sox6 expression was prominently detectable at embryonic day 12 (E12) in mitotic nuclei (Figure 2C-a (-ve control); Figure 2C-b (Sox6 antibody). Sox6 was expressed in the sub ventricular zone (svz) in migrating neurons and in the cortical plate in embryonic day 16.5, which indicates that Sox6 was primarily expressed in differentiating neurons in the cortical plate and in actively dividing neuronal precursor cells that populate the sub ventricular zone (Figure 2C-c). Sox6 was not detected in the adult brain (data not shown).

### Sox6 is a phosphoprotein

**Figure 3:**
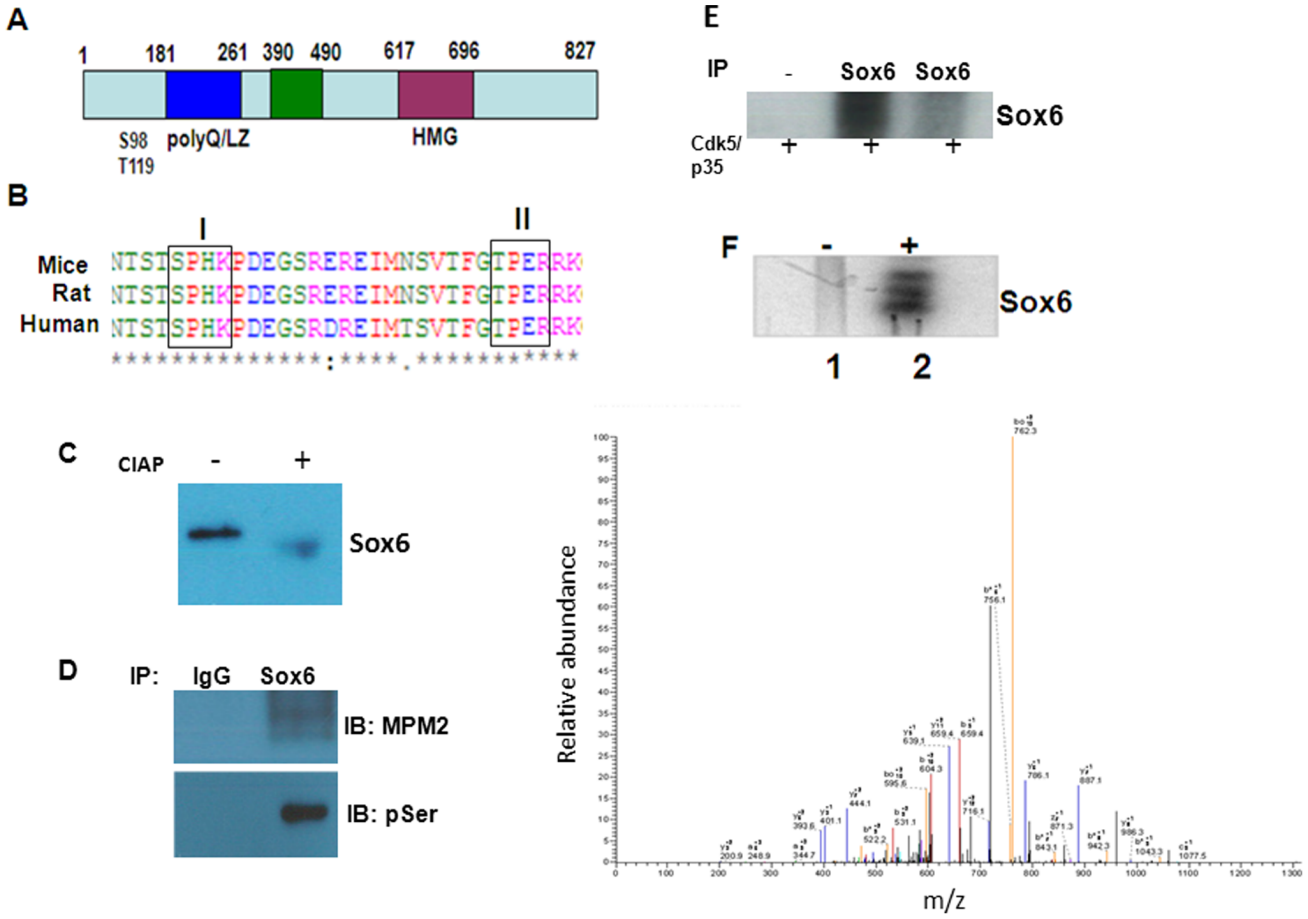
Sox6 is a phosphoprotein and a substrate of Cdk5. (A) A schematic representation of domains of Sox6 protein. Domain structure of Sox6 protein reveals that Sox6 is a phospho protein with potential Cdk5 phosphorylation sites (Ser 98 and Thr119). (B) Alignment of human, mouse, and rat Sox6 amino acid sequences. The conserved Cdk5 phosphorylation sites [(S/T)PX(K/H/R)], Ser 98 and Thr119 are boxed. The Cdk5 consensus phosphorylation site is conserved among all three species. (C) Rat brain lysate from postnatal day 1 (PN1) was subjected to calf intestinal alkaline phosphatase (CIAP) treatment followed by Western blot analysis with Sox6 antibodies. CIAP treatment resulted in faster migration of Sox6, suggesting that Sox6 is a phosphoprotein. *Lane 1*, untreated, *Lane 2*, CIAP treated lysate (D) Sox6 was immunoprecipitated from postnatal day 1 and subjected to Western blot analysis with phospho-Ser and MPM2 (phospho-Ser/ThrPro) antibodies. Lysates with no antibody immunoprecipitation was used as control. (E) Sox6 protein immunoprecipitated from postnatal day 1 (PN1) and subjected to phosphorylation assay with recombinant Cdk5/p35 (*Lanes 1, 2 and 3*). Immune complex Cdk5 assay with no antibody does not show Sox6 phosphorylation (*Lane 1*). The Cdk5 inhibitor roscovitine was added in the reaction mixture inhibited the Sox6 phosphorylation by Cdk5 (*Lane 3*). (F) The GST-Sox6 was phosphorylated with Cdk5/p35 (*lane 2*). *Lane 1* shows the negative control (without Cdk5/p35). (G) Cdk5 phosphorylates Sox6 at Thr^119^. The Sox6 protein was phosphorylated *in vitro*, digested with trypsin, and phosphopeptides were isolated using an IMAC column before separation by HPLC. Elution of the peptide T*PER (MS/MS) is shown, suggesting that Thr119 is one of the site phosphorylated by Cdk5/p35.

Cdk5 is developmentally regulated and Cdk5 knock out (KO) mice undergo developmental arrest and death (14). Based on the finding that both Sox6 and Cdk5 are developmentally regulated, we examined whether Sox6 is regulated by Cdk5. The domain structure of the full length Sox6 protein revealed that Sox6 has potential phosphorylation sites for consensus Cdk5 signature motif S/TPXR/K/H towards the N-terminal domain (Figure 3A). The Cdk5 consensus motif in Sox6 (S^98^PHK) and (T^119^PER) is conserved among human, rat and mice (Figure 3B).

We first examined whether Sox6 was phosphorylated *in vivo*. The mouse brain lysate prepared from embryonic day 18 (E18) was subjected to calf intestinal alkaline phosphatase (CIAP) treatment. Western blotting was performed using Sox6 antibodies. Sox6 showed faster migration in the CIAP treated brain lysate, suggesting that Sox6 was phosphorylated *in vivo* (Figure 3C). Next, we immunoprecipitated Sox6 protein from the brain lysate from new born mouse (PN0) and performed Western blot analysis with phospho-Ser antibody. Sox6 was detected by phospho-Ser antibodies, suggesting that it was phosphorylated *in vivo* (Figure 3D, upper panel). Likewise, Sox6 was detected by phospho-Thr and MPM2 (mitotic protein monoclonal 2, phospho-Ser/Thr-Pro) antibody (Figure 3D, lower panel). MPM2 antibody detects the proteins phosphorylated at the Ser/Thr residues followed by proline. These results confirm that Sox6 is phosphorylated *in vivo*.

### Sox6 is *in vitro* substrate of Cdk5

Because Sox6 has potential Cdk5 phosphorylation sites, we asked if Sox6 is phosphorylated by Cdk5. Sox6 immunoprecipitated from post natal day 1 (PN1) rat brain lysate was subjected to *in vitro* phosphorylation with commercially obtained Cdk5/p35. Sox6 was phosphorylated by Cdk5/p35 (Figure 3E). The Sox6 phosphorylation by Cdk5/p35 was inhibited by the Cdk5 inhibitor roscovitine, suggesting specific phosphorylation of Sox6 by Cdk5 (Figure 3E). We then examined the phosphorylation of recombinant Sox6 by Cdk5/p35. We first made GST-Sox6 recombinant construct in pGEX vector, transfected it to express GST-Sox6 and purified it. Next, we determined whether Sox6 was directly phosphorylated by Cdk5/p35. GST-Sox6 was phosphorylated *in vitro* by Cdk5/p35 (Fig. 3F). Sox6 phosphorylated by Cdk5/p35 is subjected to mass spectrometry to determine the phosphorylation sites by Cdk5/p35. The recombinant Sox6 protein was phosphorylated by Cdk5/p35 (Figure 3G) and phosphorylation sites were identified by MALDI-TOF mass spectrometry. The potential sites identified by mass spectrometry correspond to proline directed Thr residue T^119^PER (Fig. 3G). The T*PER motif is a consensus Cdk5 phosphorylation sequence. Figure 3G shows the MALDI MS spectra of Sox6 phosphorylation motif T*PER. These data suggest that Sox6 is a phospho- protein and Sox6 is phosphorylated by Cdk5.

### Sox6 associates with the Cdk5 and its Activator p35

**Figure 4:**
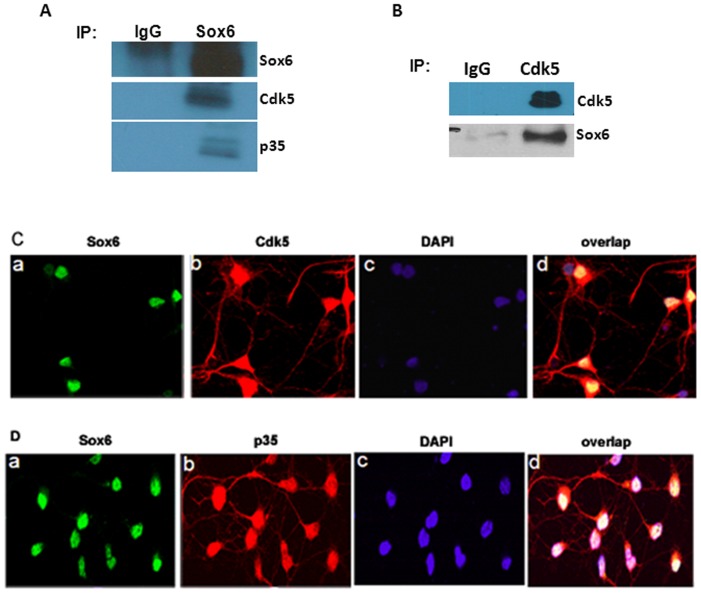
Sox6 associates with Cdk5/p35. (A) Sox6 was immunoprecipitated (IP) from postnatal day 1 (PN1) rat brain lysate and subjected to Western blotting using Sox6, Cdk5 and p35 antibodies. (B) Co-IP of Cdk5 and Sox6. The Cdk5 was immunoprecipitated from PN1 rat brain lysate and immunodetected with Sox6. (C) Colocalization of Sox6 with Cdk5. The cortical neurons were dissociated from embryonic stage 17 (E17) of rat and were cultured *in vitro* for 5 days and subjected to immunofluorescence. The Cdk5 was visualized using the monoclonal antibody (red) and Sox6 was visualized using polyclonal antibody (green). (D) Co-localization of Sox6 and p35 in nucleus. E17 dissociated rat cortical neurons were cultured *in situ* for 7 days and subjected to immunofluorescence. The Sox6 was immunostained with polyclonal antibody (green) and p35 was stained with monoclonal antibody (red). Sox6 co localizes with p35 in the nucleus.

Sox6 was tested for its ability to co-immunoprecipitate with the Cdk5/p35 complex. Lysates were obtained from wild-type mouse postnatal brain day 0 (PN0) and were subjected to immunoprecipitation (IP) with Sox6 antisera, Cdk5 antisera, or control rabbit serum. Sox6 was associated with Cdk5 and p35 as revealed by the IP of Sox6 and immunoblot analysis with anti-Cdk5 and anti-p35 antibodies (Figure 4A). Likewise, Sox6 was detected in the IP of Cdk5, but not control sera (Figure 4B). These results suggest that Sox6 was present in a complex with Cdk5 and p35.

### Sox6 co-localizes with Cdk5 and its activator p35

To examine the cellular localization of Sox6 with Cdk5/p35, immunocytochemical assays were performed on dissociated E18 rat cortical neurons cultured for 6 days. A rhodamine-conjugated secondary antibody was used to show endogenous Cdk5 and p35 expression, and FITC conjugated secondary antibody was used for endogenous Sox6 (Fig. 4C). Figure 4C shows the co-localization of Sox6 (green) with Cdk5 (red). Sox6 was expressed in the nucleus whereas Cdk5 was mainly expressed in the cell bodies, axons and dendrites. We also observed the expression of Cdk5 in the nucleus. We noticed the co-localization of Sox6 with Cdk5 in the nucleus. Figure 4D shows the co-localization of Sox6 with p35. The p35 was prominently expressed in the nucleus, cell bodies and in the axons. In the overlay image, co-localization Sox6 can be seen in the nucleus with the Cdk5 and p35. These results suggest that endogenous Sox6 colocalizes with endogenous Cdk5 and p35 in the nucleus.

### *Cdk5* ^−/−^ Mice Display Differential Sox6 expression

**Figure 5:**
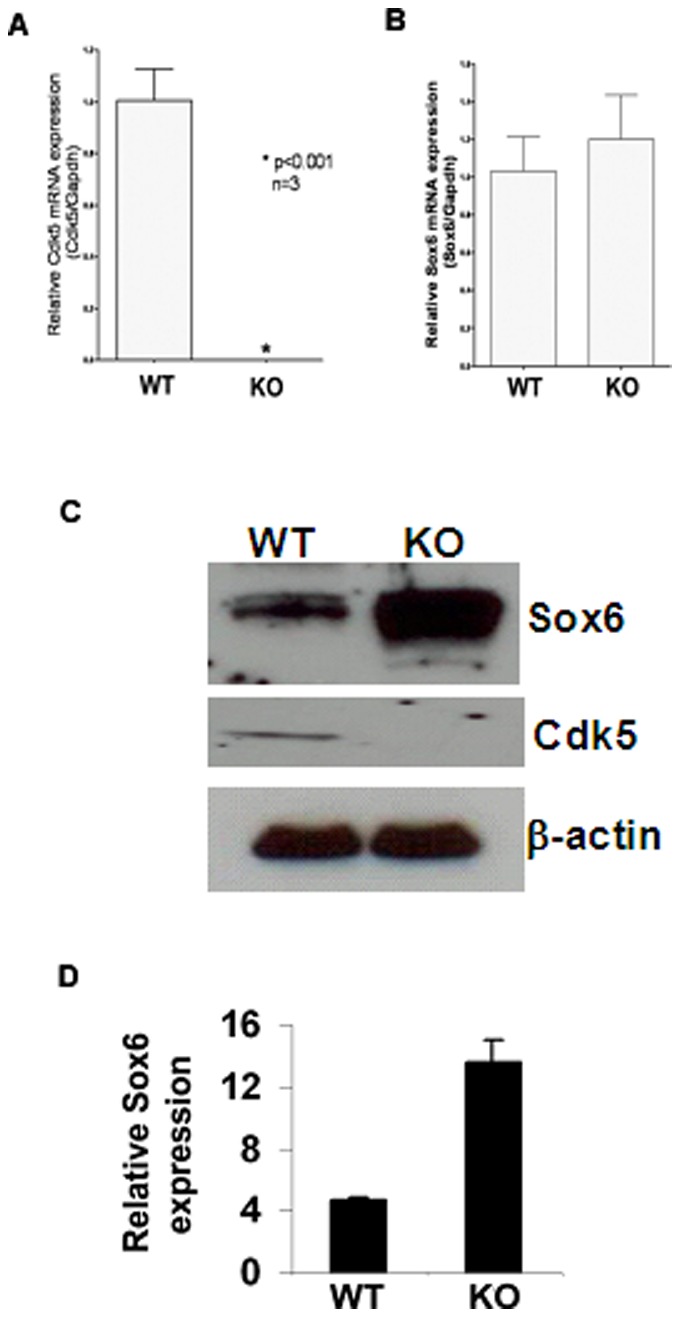
Differential steady state levels of Sox6 in WT and Cdk5^−/−^ mice. The mRNA expression of Sox6 in Cdk5 WT and Cdk5^−/−^ mouse brain. Total RNA was extracted from WT and Cdk5^−/−^ mice brain on embryonic day 17 (E17) and cDNA was prepared using reverse transcription. A) Quantitative PCR of Cdk5 expression in WT and Cdk5^−/−^ mice brain. The Cdk5^−/−^ brain shows complete abrogation of Cdk5 message. B, Sox6 expression in WT and Cdk5^−/−^ mice brain. There is no change in the Sox6 expression in WT and Cdk5^−/−^ mice n = 3 and p<0.001 (C) Lysates from embryonic day 17 (E17) age-matched wild-type (WT) and Cdk5^−/−^ mouse (KO) brain were separated by SDS-PAGE and transferred onto nitrocellulose. Cdk5 was detected with polyclonal C-8 antibody. Sox6 was immunodetected using Sox6 specific antibody. Equal loading was confirmed by immunodetection of β-actin. Cdk5^−/−^ mouse brain lysates showed elevated steady state levels of Sox6 protein. D) Densitometry analysis of Sox6 expression obtained from Figure 5C. n = 3 and *p<0.001 of Sox6 expression in Cdk5^−/−^ brain compared to WT.

In order to determine the effect of Cdk5 deficiency on Sox6 expression, we analyzed its expression in E17 Cdk5^−/−^ mice brain. We first examined the mRNA expression of Sox6 in WT and Cdk5^−/−^ brain by quantitative PCR analysis. The expression of Cdk5 was absent in Cdk5^−/−^ brain as compared to WT (Figure 5A). However, Sox6 mRNA levels remained the same in the WT and Cdk5^−/−^ brain (Figure 5B). We then examined the protein expression of Sox6 in Cdk5 WT and Cdk5^−/−^ brain lysate. The protein levels of Sox6 significantly was increased by 4-fold in Cdk5^−/−^ brains as compared to WT (Figure 5C and D). These results suggest that Sox6 expression is regulated by Cdk5.

### Inhibition of Cdk5 activity by DN Cdk5 and roscovitine increases the Sox6 expression in primary cortical neurons

**Figure 6:**
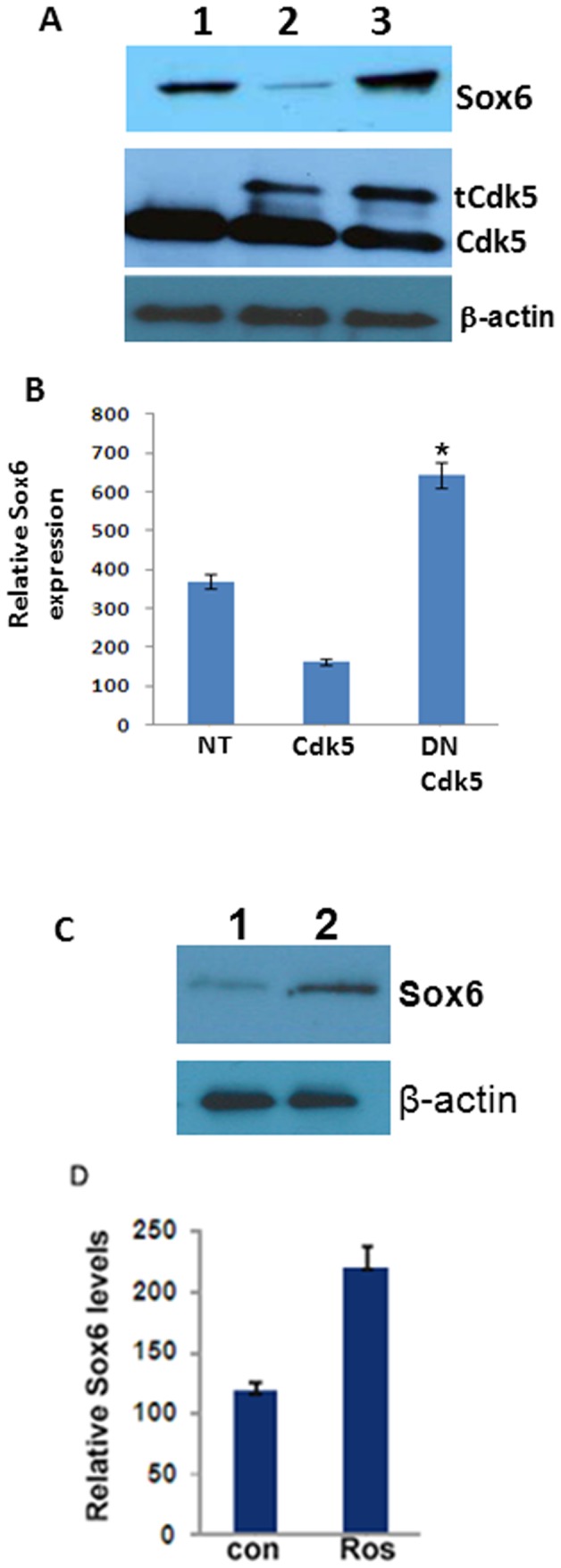
Inhibition of Cdk5 activity by DN Cdk5 increases Sox6 expression. A) We transfected the 5 DIC primary cortical neurons either with WT Cdk5 (lane 2), DN Cdk5 (lane 3) and p35 (lane 4) and then Western blot analysis was performed with Sox6 antibodies to analyze the endogenous Sox6 levels, 2-days after transfection (7DIC). Transfection of WT Cdk5 (lane 2) reduced the Sox6 levels, while transfection of DN Cdk5 (lane 3) increased Sox6 expression. The middle panel shows the transfected Cdk5 and endogenous Cdk5 levels. The lower panel corresponds to the β-actin levels. B) Densitometry analysis of Sox6 expression shown in Figure 6A. Sox6 expression compared to non-tranfected neurons, n = 3 and *p<0.01.

Next we wanted to ask if Sox6 expression is altered by the Cdk5 activity in cortical neurons. Towards this, we transfected the 5DIC neurons with Cdk5 and DN Cdk5 vectors (Figure 6A and B). Transfection of cortical neurons with WT Cdk5 reduced the endogenous Sox6 expression, whereas the transfection with DN Cdk5 increased the Sox6 expression (Figure 6A). The transfected Cdk5 (His-Cdk5) had a slightly lower mobility due to His tag. Endogenous Cdk5 was evident in all 3-lanes (lanes, 1–3) while the transfected Cdk5 (lane 2) and transfected DN Cdk5 (lane 3) showed two bands. The dominant negative Cdk5 has the same molecular weight as the WT Cdk5 except for a mutation at a single residue in the Cdk5 (D144N). The DN Cdk5 reduces the Cdk5 activity; however, Cdk5 levels remain the same, suggesting that Cdk5 activity regulate the steady state Sox6 expression. Furthermore, treatment of cortical neurons with Cdk5 inhibitor roscovitine resulted in the increase of Sox6 expression (Figure 6B). Both dominant negative Cdk5 as well as roscovitine reduced the Cdk5 activity suggesting that Cdk5 activity, regulate the steady state Sox6 expression.

## Discussion

In the current study, we provide evidence that Sox6, a developmentally regulated key transcription factor, is a phosphorylation substrate of Cdk5/p35. The co-immunoprecipitation analysis reveals a high-affinity association between Sox6 and Cdk5/p35. More importantly, Sox6 expression is developmentally regulated; it is detected in mitotic nuclei in early embryonic (E12) cortex and in migrating neurons of sub ventricular zone (svz) and cortical plate in E16. In support of this finding, Sox6 is shown to be necessary for cortical interneuron migration [8]. Sox6 expression is abrogated in the adult mouse brain. Our finding that Sox6 expression is significantly high in Cdk5^−/−^ brain suggests that mis-expression of Sox6 is regulated by Cdk5.

A number of cytoplasmic proteins have been identified as substrates of Cdk5. Cdk5 plays a multifunctional role in neurons including neurite outgrowth, cytoskeleton assembly, and synaptic transmission by phosphorylating these diverse cytoplasmic targets [12], [29]. Deregulation of Cdk5 activity mediates the hyperphosphorylation of the major cytoplasmic component of fibrillary tangle, tau, triggered by Aβ and aberrant NF-M/H phosphorylation, suggesting that Cdk5 is involved in neuronal apoptosis via a cytoplasmic mechanism [30], [31].

On the other hand, Sox6 is a transcription factor present in the nucleus. Genome-wide association studies suggest that Sox6 influences both obesity and osteoporosis [32]. Sox6 is an important enhancer of definitive erythropoiesis [33]. Pertaining to the brain, Sox6 is essential for cortical interneuron development. Sox6 is shown to be important for normal positioning and maturation of MGE-derived interneurons, and that the specific deletion of Sox6 results in epileptic encephalopathy [8]. Recently, it was found that *Sox6* is expressed and required in MGE-derived cortical interneurons, and additionally it plays an independent role in pallial/subpallial patterning [34]. Recent work by the same group has also identified *Sox5*, a close homolog of *Sox6*, as being required for the specification of deep layer pyramidal neurons in layer V and VI of the cortex [3]. miRNA 219 and miR-338 have been shown to inhibit Sox6 and block oligodendrocyte differentiation [36]. Sox6-deficient mice are anemic due to impaired red cell maturation and show inappropriate globin gene expression in definitive erythrocytes.

In contrast to classic Cdks whose activity is regulated during cell cycle by cyclins, Cdk5 is inactive in cell cycle [29], instead, its activation requires association of Cdk5 with its neuron-enriched regulators p35 or p39 [12]. *In vivo* Cdk5 activity is tightly regulated. Complete lack of Cdk5 is clearly destructive to central nervous system, with deletion of either p35 or Cdk5 resulting in profound neurological defects [37], [38] and Cdk5^−/−^ mice die *in utero*. Cdk5 hyperactivity is toxic to cells, particularly neurons, leading to neuronal apoptosis under either physiological or pathological conditions [39], [40]. Neurotoxicity deregulates Cdk5 activity either through generating a more stable proteolytic product of p35, p25, or by stabilizing Cdk5/p25 complex. Transgenic p25 mice display neuronal loss and cognitive impairment [41]. Furthermore, Cdk5/p25 deregulates HDAC1 during neurotoxicity [20].

Cdk5 has been proposed to play a key role in neurodegenerative diseases primarily through a cytoplasmic mechanism. Both Cdk5 and p35 are enriched in the cytoplasm of neurons where Cdk5 phosphorylates various substrates including tau, a major component of neurofibrillary tangles associated with neurodegeneration [31]. Interestingly, several studies including this, show that Cdk5 and p35 are localized in the nucleus [42]. Sox6 appears to have multiple functions in the brain. Sox6 is expressed more in an early embryonic stage and gradually reduces during the development in to an adult. In conclusion, this study highlights the role of Cdk5 in nucleus regulating developmental transcriptional factor Sox6.
